# The College Students’ Sense of Responsibility for Innovation and Entrepreneurship

**DOI:** 10.3389/fpsyg.2020.02049

**Published:** 2020-08-26

**Authors:** Qing Zhang, Congchong Liu, Zehao Wang, Zimo Yang

**Affiliations:** ^1^Nanjing Xiaozhuang University, Nanjing, China; ^2^School of Business Administration, Wonkwang University, Iksan, South Korea; ^3^Shandong Technology and Business University, Yantai, China; ^4^Graduate School of Pan-Pacific International Studies, Kyung Hee University, Seoul, South Korea

**Keywords:** innovation and entrepreneurship, social responsibility, positive psychological quality, linear regression analysis, factor analysis model

## Abstract

The purpose of this paper is to analyze the relationship between positive psychological quality and college students’ sense of responsibility for innovation and entrepreneurship from the perspective of positive psychology, to explore the cultivation model that can effectively improve college students’ sense of responsibility for innovation and entrepreneurship, and to promote their success in entrepreneurship. In this study, a total of 1,500 college students were selected for questionnaire survey. ANOVA was used to analyze the differences of innovation and entrepreneurship responsibilities in demographic variables; factor analysis models were used to explore the factors that influence college students’ sense of responsibility for innovation and entrepreneurship; and Spearman correlation and linear regression were used to analyze the relationship between college students’ positive quality and innovation and entrepreneurship. The results showed that the average scores of individual responsibility, team responsibility, and social responsibility were 3.290, 3.624, and 3.720, respectively; individual responsibility differed significantly at the grade level; group responsibilities and social responsibilities were significantly different at the grade and gender levels; the linear fitting between benevolence, super-excellence, bravery, restraint, and wisdom with team responsibilities all reached significant levels, among which the wisdom coefficient was the highest; the linear fitting between syngroup, excellence, bravery, modesty, and wisdom with social responsibility reached a significant level, among which the wisdom coefficient was the highest; the linear fitting between syngroup, excellence, bravery, modesty, and wisdom with personal responsibility reached a significant level, among which the coefficient of excellence was the highest. This indicated that positive psychological qualities such as syngroup, excellence, modesty, benevolence, super-excellence, bravery, restraint, and wisdom were the influencing factors of college students’ sense of responsibility for innovation and entrepreneurship. Among them, the role of wisdom is the most noteworthy in predicting social and group responsibilities, and super-excellent is the most significant predictor for individual responsibility.

## Introduction

Entrepreneurship of college students is an entrepreneurial process that takes a special group of college students and graduates as entrepreneurs (Ali et al., 2019). With the continuous transformation process of China in recent years and the increasing pressure of social employment, entrepreneurship has gradually become a career choice for college students and graduates (Qiao and Huang, 2019). However, the current college entrepreneurship education system in China is imperfect, and it has no practical application value for cultivating undergraduates’ sense of entrepreneurial responsibility and improving their innovative ability. Relevant research showed that the core problem faced by current college students was not only the ability to take on financial risks but also the psychological responsibility to withstand setbacks (Obschonka et al., 2018). Although college students are keen on entrepreneurship, they lack self-awareness by blindly following the trend of entrepreneurship and using entrepreneurship as a means of comparison and competition, regardless of whether they are suitable for entrepreneurship or whether they are mature enough for entrepreneurship. This kind of college students’ entrepreneurial psychology is utilitarian, accompanied by fear of difficulties and lack of responsibility, which is an unhealthy psychological problem (Sang and Lin, 2019). Therefore, it is extremely necessary to cultivate college students’ sense of entrepreneurial responsibility and improve their innovative ability.

College students need entrepreneurial motivation for their own entrepreneurship, and the foundation of entrepreneurial motivation is guaranteed by excellent psychological quality and sound personality. Entrepreneurial psychological quality is a discussion of psychological quality at the entrepreneurial level, that is to say, a psychological ability aimed at innovative entrepreneurial behavior established by college students through the influence of acquired learning and educational environment in the entrepreneurial process (Al-Jubari et al., 2019). Relevant research showed that when individuals faced entrepreneurial problems, they would show different psychological cognitive response behaviors, which is an effective comprehensive psychological quality that can enhance the ability of entrepreneurs. Therefore, the psychological factors behind the entrepreneurial behavior of college students are also the focus of research by many scholars. For example, positive psychology has gradually become a hot spot in current psychology research, and its core concept is to mobilize the individual’s internal psychological factors, such as self-esteem, honesty, happiness, and bravery. With the help of positive psychological qualities, new rules are hoped to be figured out for interpretation (Baluku et al., 2019). If college students encounter setbacks and difficulties in entrepreneurship, positive psychology can help them solve problems, avoid risks, and achieve ultimate success (Tang, 2019).

To sum up, it has become a research trend to enhance the sense of entrepreneurial innovation responsibility of contemporary college students and improve the ability of innovation and entrepreneurship, and good psychological quality of entrepreneurship is indispensable in the process of entrepreneurship of college students. Therefore, this study explored the status quo and influencing factors of college students’ sense of responsibility for innovation and entrepreneurship from the perspective of positive psychology.

## Literature Review

### Interpretation of College Students’ Sense of Responsibility

In academic circles, different subjects have different interpretations of college students’ sense of responsibility. In sociology, the concept of sense of responsibility is discussed as “When individuals or groups want to contribute to national construction and social maintenance through their own efforts, they show their high quality in the process. The sense of responsibility is not produced in a plain way, it is formed in the social values and a good atmosphere. In other words, it is the correct understanding of the identity given by the society by individuals or groups, and on top of this understanding, it is necessary to take action for it” (Yang et al., 2012). From the psychological level, the sense of responsibility refers to the individual’s moral level to a certain level, in the process of self-awareness development of their own status and identity. The emotional changes are generated under the goal of achieving moral consciousness in participating in social activities. Responsibility comes from the change of psychology and thought (Hill and Torres, 2010), but there are obvious differences on the specific content of the psychological structure of responsibility, and the specific conclusions of each dimension are different. Some experts and scholars regard the sense of responsibility of college students as the psychological change process of college students, the awakening of self-consciousness caused by the stimulation of the external environment, and the domination of thoughts and actions under the understanding of responsibility (Wei and Feng, 2013).

### The Connotation and Forming Factors of College Students’ Sense of Responsibility

Some scholars have analyzed the position of college students. They think that as adults who have reached the age of 18, college students will have the ability to be responsible for themselves, family, and society. Especially as an adult, he/she should fulfill the tasks given by the society and make his/her own contribution to the society. Under this understanding, they equate the sense of responsibility of college students with their sense of social responsibility. The research and discussions on college students’ sense of responsibility in specific social roles and tasks have also been carried out in the academic community. College students’ sense of family responsibility is a sense of responsibility that college students should have, and it is also an important part of social responsibility (Yin, 2018). First of all, an adult should make sure that he/she is no longer a child in the family, and thus he/she should assume the family responsibilities according to the adult’s standard and learn how to restrain behaviors and abide by the family rules, so as to maintain the harmonious development of family. The changes in psychology and actions formed under this recognition reflect the awakening of college students’ sense of family responsibility (Wang, 2014). In addition, in terms of learning, college students should be more self-conscious and actively learn skills and knowledge. In order to meet the needs of future work and their own development, when college students can consciously complete the learning tasks and achieve the learning goals, it indicates that their sense of responsibility for learning has also formed. Learning responsibility consists of three parts: learning responsibility, learning responsibility, and responsible behavior (Chen and Shen, 1993). It is an integral part of students’ moral character. Besides, learning responsibility urges individuals to form strong learning motivation and then urges them to actively make continuous efforts to overcome difficulties and solve problems (Zhao, 2019). In a word, college students are willing to learn, consciously complete their learning tasks, and fulfill their student obligations, thereby enabling the formation of college students’ sense of responsibility for learning (Liu, 2017). With regard to college students’ sense of responsibility for innovation and entrepreneurship, college students’ sense of responsibility refers to the social relations and the awareness of personal ability to undertake duty or corresponding negligence under the background of “mass entrepreneurship and innovation” (Ai, 2017). At the same time, from the perspective of technological design philosophy, responsible innovation also includes a kind of professional responsibility to some extent. Some studies have shown that college students’ sense of responsibility can be divided according to their living environment, social roles, and responsibilities. Therefore, the sense of responsibility for innovation and entrepreneurship is that under the influence of the external environment, college students have formed the necessary sense of innovation and entrepreneurship, developed the spirit of innovation and professionalism, and actively innovated and ventured boldly. Under the motivation of innovation and entrepreneurship, to promote individuals to actively complete the task of entrepreneurship and innovation, according to their social relations, should include innovation and entrepreneurship self-responsibility, group responsibility, and social responsibility. For the purpose of building the sense of responsibility, college students need to achieve “four unities.” The first step is to correctly recognize their own responsibilities, and then form the emotion of fulfilling their responsibilities, and then to further strengthen the awareness of fulfilling their responsibilities. The last step is to put the awareness into the action of time. Each process cannot be separated from the foundation of the previous process. Those four factors shall work together and finally promote college students to form a sense of responsibility (Li and Geng, 2010). College students’ inadequate sense of responsibility can be reflected in many aspects. Therefore, the problem of influencing factors of college students’ sense of responsibility has always been the focus of experts in various fields. Some studies have examined demographic characteristics, such as the differences in gender and family background (Huang et al., 2016; Wu and Wang, 2018), personality characteristics (Xue et al., 2016), and interpersonal relationship (Chen, 2013).

### Innovation and Entrepreneurship Education

As world trends continue to change, China’s economic system reform will also undergo transformation. Nowadays, under the general trend of the world, China’s industrial type will change from labor intensiveness to core technology. At this stage, it is necessary to focus on cultivating the innovative thinking of the public and improving the entrepreneurial ability. In the process of China’s economic system transformation, it is concerned about the new generation, especially college students in the conceptual stage of career development. On December 28, 2016, Renmin University of China released the *2016 China Student Entrepreneurship Report* (hereinafter referred to as “the Report”). Through a questionnaire survey of 1,767 college graduates in China, the report found that college students in China had a high enthusiasm for entrepreneurship. At the same time, according to the report released in 2017–2018, the entrepreneurship rate of undergraduate graduates was higher than that of graduate students and junior college graduates in terms of academic qualifications; from the perspective of university type, the entrepreneurship rate of ordinary undergraduates was higher than that of the undergraduates with double first-class construction; the top three in terms of entrepreneurship industry in terms of college students culture, sports and entertainment, education, wholesale, and retail are in order. From the perspective of the types of entrepreneurial projects, the top three include business services, education and training, and cultural creativity, which has set off a wave of innovation and entrepreneurship among college students across the country, also made colleges and universities provide theoretical support and help for students (Rogoza et al., 2018; Sun, 2020).

At present, China pays great attention to the innovation and entrepreneurship education of college students. Through the research of crime investigation and other ways, it is obvious that there are still many defects in the innovation and entrepreneurship education of college graduates. For example, for the moral education of college students, it is necessary to advocate to infiltrate into the innovation and entrepreneurship education of students from various channels, focusing on its education of sense of responsibility. In addition, there are still many problems in the cultivation of students’ innovation and entrepreneurship awareness and ability (Liu et al., 2019). Individual value includes not only economic value but also humanistic value and self-realization value. Entrepreneurship education embodies the economic value of entrepreneurship education, but the humanistic value and spiritual value of entrepreneurship education are the most fundamental. Entrepreneurship education focuses on cultivating students’ innovation and entrepreneurship ability, moral awareness, enhancing students’ sense of personal dignity, and enhancing their own value awareness. When studying the innovation and entrepreneurship education of college graduates, researchers should not only combine the actual situation of China but also learn from the experience of other countries (Yu et al., 2020). For instance, the United Kingdom has made a lot of achievements in its entrepreneurship education, and many inspirations can be obtained; in the process of innovation and entrepreneurship education, it is necessary to first create a good policy environment, which needs the support of the government; secondly, it is necessary to improve the infrastructure of entrepreneurship education; finally, China needs the full support and help of the society for the innovation education of colleges and universities. These theoretical research results have laid a foundation for the solution of the problem of improving the sense of responsibility of college students. On the basis of the existing research results, some scholars have carried out theoretical discussion (Sun et al., 2015) and path analysis on the cultivation of the sense of responsibility in the innovation and entrepreneurship education of college students. However, there is a lack of discussion on the influencing factors and mechanism of college students’ responsibility for innovation and entrepreneurship, and the specific research on college students’ innovation and entrepreneurship responsibility from the perspective of positive psychology has not yet appeared.

### About the Positive Psychology

Among many factors that affect college students’ sense of responsibility for innovation and entrepreneurship, psychology is an important predictor. Positive psychology is a new psychological concept emerging in western countries at the end of the last century. It aims to stimulate people’s initiative and innovation ability by mining positive energy, so as to achieve a happier life. Foreign countries have made some achievements in the criticism and reflection of positive psychology. About positive experience, foreign scholars have discussed subjective well-being (Daniel Kahneman and Taylor benshahar), optimism (Peter), positive emotions, and physical health (Dctweeler and Steward). In terms of positive psychological quality, this paper discusses the psychological mechanism and neurobiological basis of hope and optimism, summarizes the five factor theories of personality traits (Alan CARR), and creates the positive psychological quality assessment scale (Lopez and Snyder, 2004). In terms of the influence of positive psychology on human behavior, scholars have come to different conclusions through different methods: Sean Ekor has passed through 48 countries. The data of 2,000 people and 225 subtopics strongly prove that happiness can bring great changes to organizations and individuals: the average increase of organizational productivity was by 31%, the average increase of customer satisfaction by 12%, the increase of work efficiency by 16%, the increase of work engagement by 32%, and the increase of work satisfaction by 46% (Matin and Christopher, 2004); Angela Dukeworth found that in order to succeed, the researchers needed a very important factor, that is, perseverance; Kelly Magnegal put forward the theory of limit of willpower loss, was opposed to compulsive therapy, advocated the effective use of their desires, and turned their guidance into the motivation to focus on certain things or ignore the temptation. This paper summed up a healthy life mentality, that is, to dare to face challenges (Sun et al., 2015). Besides, on the basis of the possibility and biological limitations of change, Dr. Seligman found that focusing limited time and energy on traits that could be changed was the most effective way to achieve self-improvement (Huang et al., 2018).

### Positive Psychological Quality and Innovation and Entrepreneurship

Previous studies have shown that people with strong adaptability have strong innovation tendency, low sense of responsibility, high altruism tendency, and strong entrepreneurial tendency (Zhao et al., 2018); positive psychological quality is the positive psychology formed by the interaction between individuals and their surrounding environment in the process of growth. The expression of experience and emotion as well as the optimistic attitude toward problems have a very important impact on college students who are about to enter the society for entrepreneurial practice; college students’ innovation and entrepreneurship need to correctly handle team relations, fulfill team obligations, and assume team responsibilities. Positive psychological quality has a significant positive correlation with freshmen adaptation and self-awareness and has a significant negative correlation with interpersonal problems (Halvari et al., 2019); the willpower of positive psychological quality is an important factor in time monitoring and time efficiency. College students’ sense of responsibility for innovation and entrepreneurship includes effective planning and implementation (Ye, 2020); it is speculated that there may be a certain correlation between college students’ positive psychological quality and their sense of responsibility for innovation and entrepreneurship.

## Materials and Methods

### Design of Questionnaire

With the college students’ sense of responsibility for innovation and entrepreneurship and the influence of positive psychological quality on it, a special scale for college students’ sense of responsibility for innovation and entrepreneurship was designed based on Professor Martin Seligman and Christopher questionnaire of positive psychological quality compiled by Professor Peterson and the questionnaire of 2016 college students’ positive psychological quality compiled by Associate Professor Chen Wanling; based on Zhuo Jin’s work responsibility in 2006 and Wang Yinghui’s entrepreneurship ability questionnaire in 2018, a special scale of college students’ innovation and entrepreneurship responsibility was designed and a questionnaire of college students’ innovation and entrepreneurship responsibility was compiled (Wang and Tan, 2020).

The questionnaire includes three parts: basic personal situation, college students’ positive psychological quality, and their sense of responsibility for innovation and entrepreneurship. The basic information includes “gender,” “the only child situation,” and “grade.” The purpose of this study is to understand the individual factors of college students’ innovation and entrepreneurship responsibility. The main part of the questionnaire focuses on the current situation and positive psychological quality of college students’ sense of responsibility for innovation and entrepreneurship. The survey of college students’ sense of responsibility for innovation and entrepreneurship was designed from three aspects: personal responsibility, team responsibility, and social responsibility. There were 15 questions in total to explore college students’ understanding of related responsibilities, to determine their positive identification of responsibility events, and to judge the strength of implementation of responsibility behaviors (Tok, 2020). The survey of college students’ positive psychological quality was conducted based on 24 excellent qualities of positive psychological quality, with 32 items in total. In the main part of the questionnaire, five Likert scales were used to score each item, and the average score of the measurement item was used to express the degree of the respondents’ sense of innovation and entrepreneurship responsibility. The higher the score, the higher the responsibility of innovation and entrepreneurship. Among them, 1 point means *strongly disagree*, 2 points means *disagree*, 3 points means *neutral*, 4 points means *agree*, and 5 points means *strongly agree* (Princitta and Menachery, 2020).

### Sample and Data Collection

In this study, college students from eight universities in Fujian, Jiangsu, Anhui, Heilongjiang, and Zhejiang were selected as the research objects. There was no immoral behavior in the research process, and no human clinical or animal experiments were involved. The authors first got in touch with the counselors of various colleges and then asked if the survey could be conducted in schools. With the approval of the counselor, a total of 1,511 questionnaires were distributed. This questionnaire was answered anonymously. A total of 1,511 questionnaires were collected, 11 invalid questionnaires were eliminated, and 1,500 were valid.

### Control of Virtual Variables

This study focuses on the influence of positive psychological quality on the sense of responsibility for innovation and entrepreneurship. Previous studies have shown that different demographic characteristics may have an impact on the sense of responsibility. Therefore, demographic variables were selected as control variables. In this data analysis, gender and the only child situation were encoded as virtual variables.

### Statistical Analysis

SPSS 19.0 version statistical software was used to analyze the data processing of this study, the measurement data were expressed by mean ± standard deviation ($\bar{x}\pm s$), and the count data were expressed as a percentage (%); one-way ANOVA was used to analyze the difference of innovation and entrepreneurship responsibilities in demographic variables (grades); multivariate analysis of variance was used to examine the differences in innovation and entrepreneurship responsibilities in demographic variables (grades); a factor analysis model was used to explore the factors that influence college students’ sense of innovation and entrepreneurship responsibilities. In addition, Spearman correlation and linear regression were used to observe the relationship between college students’ positive quality and the responsibility of innovation and entrepreneurship. Lastly, Origin8.0 software was used to draw the diagram.

## Analysis and Results

### Sample Analysis of College Students

#### General Information and Sample Validity of College Students

In this study, descriptive statistical analysis was made on the basic characteristics of the respondents from three aspects: gender, the only child situation, and university grade. The specific analysis results are shown in Table 1. Among the 1,500 valid samples collected, 685 were men, accounting for 45.7%; and 815 were women, accounting for 54.3%. The proportion of men and women was roughly the same, indicating that this survey can show men and women in a more balanced way. The respondents’ sense of responsibility for innovation and entrepreneurship can also balance the difference between male and female respondents’ sense of responsibility for innovation and entrepreneurship, so the sample has a strong representative nature; secondly, 68.6% of the respondents were not an only child, and they all had brothers and sisters, including 238 for male-only children, 244 for female, 458 for male not an only child, and 571 for female, basically 1:1. In grade distribution, the largest proportion of freshmen was 33.4%, sophomores 25.67%, junior students 16.67%, and senior students 24.27%. According to Table 2, the Kaiser–Meyer–Olkin (KMO) value of the sample sufficiency of college students’ innovation and entrepreneurship responsibility is 0.943, which indicates that the survey of college students’ innovation and entrepreneurship responsibility in the third part of the questionnaire is scientific and reasonable with high credibility.

**TABLE 1:** The basic situation of the survey object.

Characteristics	Categories	Male	% of sample male	Female	% of sample female
Only child situation	Yes	238	34.74	244	29.94
	No	447	65.26	571	70.06
Grade	Freshmen	228	33.28	273	33.51
	Sophomores	182	26.57	203	24.90
	Junior students	107	15.62	143	17.54
	Senior students	168	24.52	196	24.05

**TABLE 2:** Sample validity.

Kaiser–Meyer–Olkin measure of sampling adequacy		Part 2
		0.943
Bartlett’s test of sphericity	Approx. chi square	10,860.111
	Df	105
	Sig.	<0.001

#### Sample Mean and Covariance

Table 3 shows the overall and subdimensions of the sample of this study. The total average score of college students’ sense of responsibility for entrepreneurship and innovation is 3.545, and the overall degree of compliance is in the general to consistent range. Most of the students have a sense of team, self, and social responsibility. According to the statistical results, it is obvious that the average scores of individual responsibilities, team responsibility, and social responsibility are 3.290, 3.624, and 3.720 respectively, among which social responsibility > team responsibility > individual responsibility. The overall level of college students’ sense of responsibility for innovation and entrepreneurship is not high. According to the survey results, the overall scores of college students’ entrepreneurship and innovation responsibility were only at a general level; and the average scores of college students’ individual responsibility, team responsibility, and social responsibility were all less than 4, which indicated that the overall sense of entrepreneurial innovation responsibility of college students was low.

**TABLE 3:** Responsibility of innovation and entrepreneurship of college student.

Population covariance				

Item	Min	Max	Average value	Std. deviation
Q1	1	5	3.7140	0.66169
Q2	1	5	3.5820	0.70872
Q3	1	5	3.7700	0.73946
Q4	1	5	3.3240	0.80547
Q5	1	5	3.7640	0.67077
Q6	1	5	3.6773	0.76699
Q7	1	5	3.8387	0.78041
Q8	1	5	3.5113	0.80807
Q9	1	5	3.4613	0.81216
Q10	1	5	3.3800	0.77287
Q11	1	5	2.9667	0.92779
Q12	1	5	3.4900	0.72359
Q13	1	5	3.7587	0.79719
Q14	1	5	3.5967	0.74186
Q15	1	5	3.7640	0.71878
Team responsibility	1	5	3.6236	
Social responsibility	1	5	3.7200	
Individual responsibility	1	5	3.2901	

#### The Status Quo of College Students’ Sense of Responsibility for Innovation and Entrepreneurship

Western scholar Wilson proposed that “rationality and behavior are necessary and inseparable to a moral action, just like two sides of a coin. The behavior without reason cannot be called moral behavior. Similarly, the operation without morality must not be moral with only moral reason or reason.” Moral cognition and moral behavior are equally important. The combination of knowledge and action is the highest level of personal moral cultivation. However, the survey results indicate the average score of college students is only 2.9667 when it comes to “I have a certain entrepreneurship or innovation plan (Q11),” which shows that most of them have not yet started to make entrepreneurship and innovation plans. As for the item “I think innovation spirit is necessary for every college student (Q7),” the average score of college students is 3.8387; that is to say, college students’ sense of responsibility for innovation and entrepreneurship has a certain degree of different knowledge and behavior, high knowledge, and low behavior, which shows that college students’ cognition and emotional identification of their responsibility for innovation and entrepreneurship are clear to some extent. However, there is a lack of self-discipline and initiative in the level of responsible behavior in real life.

### Analysis on the Difference of Demographic Variables of the Responsibility of Innovation and Entrepreneurship

#### Difference Analysis of Personal Responsibility in Demographic Variables

As shown in Table 4, there was no significant difference in personal responsibility in terms of gender, whether or not the only child, and the gender of the only child (*p* > 0.05); however, there was a significant difference in personal responsibility at the grade level (*p* < 0.05), among which the personal responsibility of fourth-grade students was significantly higher than that of other grades, and the personal responsibility of third-grade students was significantly higher than that of first-grade and second-grade students.

**TABLE 4:** An analysis of the difference of personal responsibility in general demographic variables.

Variables	Classification	Personal responsibility	The value of χ^2^	The value of *p*
Gender	Male	3.311 ± 0.357	2.655	0.739
	Female	3.286 ± 0.419		
Grade	First grade	2.736 ± 0.258	6.771	0.026
	Second grade	2.675 ± 0.509		
	Third grade	3.299 ± 0.380		
	Fourth grade	3.417 ± 0.492		
Whether or not the only child	Yes	3.269 ± 0.533	7.276	0.066
	No	3.331 ± 0.275		
The only child	Male	3.431 ± 0.631	3.269	0.071
	Female	3.265 ± 0.447		

#### Analysis on the Difference of Group Responsibility in Demographic Variables

As shown in Table 5, there was no significant difference in group responsibility at the only child level (*p* > 0.05); there were significant differences in group responsibilities at the grade level (*p* < 0.05). Among them, the group responsibilities of the fourth-grade students were significantly higher than those of other grades, and the group responsibilities of the third-grade students were obviously higher than those of the first- and second-grade students; there were significant differences in group responsibility at the gender level and the one-child gender level (*p* < 0.05), among which the group responsibility of men was significantly higher than that of women.

**TABLE 5:** Analysis of the difference of group responsibility in general demographic variables.

Variables	Classification	Group responsibility	The value of χ^2^	The value of *p*
Gender	Male	3.831 ± 0.662	1.805	0.026
	Female	2.592 ± 0.470		
Grade	First grade	2.370 ± 0.472	5.776	0.021
	Second grade	2.491 ± 0.729		
	Third grade	3.511 ± 0.498		
	Fourth grade	3.802 ± 0.558		
Whether or not the only child	Yes	3.229 ± 0.418	2.686	0.043
	No	3.379 ± 0.736		
The only child	Male	4.112 ± 0.574	7.045	0.015
	Female	2.791 ± 0.662		

#### Difference Analysis of Social Responsibility in Demographic Variables

As shown in Table 6, there was no significant difference in social responsibility at the only child level (*p >* 0.05); there were significant differences in social responsibility at the grade level (*p <* 0.05), among which the social responsibility of fourth-grade students was significantly higher than that of other grades, and the social responsibility of third-grade students was significantly higher than that of first- and second-grade students; there was a significant difference in social responsibility between the sexes and the gender of the only child (*p <* 0.05). Among them, the social responsibility of men was significantly higher than that of women.

**TABLE 6:** An analysis of the difference of social responsibility in general demographic variables.

Variables	Classification	Social responsibility	The value of χ^2^	The value of *p*
Gender	Male	3.953 ± 0.460	6.805	0.018
	Female	2.366 ± 0.572		
Grade	First grade	2.283 ± 0.311	5.171	0.036
	Second grade	2.347 ± 0.279		
	Third grade	3.766 ± 0.338		
	Fourth grade	4.022 ± 0.472		
Whether or not the only child	Yes	3.209 ± 0.442	4.686	0.063
	No	3.430 ± 0.381		
The only child	Male	3.882 ± 0.157	6.045	0.032
	Female	2.744 ± 0.266		

### An Analysis of the Factors Influencing College Students’ Sense of Innovation and Entrepreneurship

#### The Selection of the Influencing Index of College Students’ Sense of Innovation and Entrepreneurship

The factor analysis model of college students’ sense of responsibility for innovation and entrepreneurship was built. According to the model and method of factor analysis, factor analysis was conducted on the data of 1,500 college students’ psychological quality influencing factors. The test steps were as follows.

*Indicator Selection*: The factors influencing college students’ sense of responsibility for innovation and entrepreneurship are as follows: X1, “I am sure that I can accomplish a new task”; X2, “I always try my best to deal with difficulties”; X3, “I am good at learning new knowledge”; X4, “I can admit my imperfections and accept them”; X5, “I am said to be confident by others”; X6, “I often offend people unintentionally”; X7, “I like to accept new challenges”; X8, “I often regret doing things carelessly”; X9, “When angry, I will restrain myself”; X10, “I will sincerely appreciate helping others”; X11, “I’m good at mobilizing everyone’s enthusiasm and enthusiasm”; X12, “It is not easy for me to get revenge”; X13, “I get very sad when I see others in a difficult situation”; X14, “Others say I’m a good partner”; X15, “I’m always energetic”; X16, “People often say that my advice is very good”; X17, “I am always interested in new things”; X18, “I never participate in class activities that I don’t like”; X19, “People say that I’m very stubborn”; X20, “I am more likely to complete tasks with you than to do it alone”; X21, “I’m willing to listen to others’ opinions”; X22, “In my life, I can find many people and things to be grateful for”; X23, “Even if the task is difficult, I will not give up”; X24, “I appreciate the beautiful things”; and X25, “When I encounter difficulties, someone will help me.”

According to the principle of factor analysis, the SPSS software was used to process the collected sample data. The SPSS software was used to analyze the factors influencing the psychological quality of college students’ sense of responsibility for innovation and entrepreneurship, and the main components were obtained and renamed.

#### Factor Analysis Process of Influencing Index of University Students’ Sense of Innovation and Entrepreneurial Responsibility

The process of factor analysis deals with utilization indexes: standardize the original data. The z-scores method is used to divide the known variables or related indicators from the mean value and the standard deviation. After standardization, the average value is 0, and the standard deviation value is 1. By judging KMO and Bartlett’s sphere, researchers can test whether the variables can meet the actual needs of unfolding factor analysis. As shown in Tables 7, 8, the KMO value was sufficient for the questionnaire sample, which means that positive psychology reaches 0.894, while the significance probability of chi square statistical value is less than 0.001, and the results show that it reaches a significant level; so it can be concluded that due to the influencing factors of positive psychology, there is a significant correlation between all the variables associated with it, showing that it can meet the needs of factor analysis. The SPSS software was used to expand factor analysis for sample data, the characteristic equation and the score of principal component factor can be obtained by operation, and the factor contribution rate can be obtained on this basis. When the factor is selected, the basic principle with eigenvalue greater than 1 should be followed. After that, the principal components can be obtained by orthogonal rotation of the factor load matrix. The obtained contents are shown in Table 9. In the table, it is not difficult to find that the number of factors extracted by factor analysis is 6, which can explain 60.924% of the total variance of the project.

**TABLE 7:** Sample validity.

Kaiser–Meyer–Olkin measure of sampling adequacy		Part 1
		0.894
Bartlett’s test of sphericity	Approx. chi square	14,084.429
	Df	300
	Sig.	<0.001

**TABLE 8:** Variance interpretation of scale samples.

	Total	Percentage of variance	Accumulate %
Part 1	3.538	14.15	14.15
	2.855	11.42	25.57
	2.849	11.395	36.966
	2.314	9.255	46.221
	2.054	8.217	54.438
	1.617	6.467	60.905

**TABLE 9:** Factor load matrix after rotation.

Part 1	Component					
	
	1	2	3	4	5	6
X12	0.775					
X10	0.734					
X24	0.710					
X1	0.693					
X15	0.633					
X22	0.602					
X4		0.809				
X2		0.807				
X5		0.782				
X7		0.756				
X23		0.754				
X9			0.782			
X8			0.677			
X21			0.566			
X19			0.564			
X14				0.670		
X6				0.618		
X16				0.570		
X3				0.566		
X17				0.512		
X11					0.841	
X18					0.782	
X20					0.777	
X13						0.877
X23						0.850

Principal component 1 is named excellence by X12, X10, X24, X1, X15, and X22.

Principal component 2 is named brave by X4, X2, X5, X7, and X23.

Principal component 3 is named restraint by X9, X8, X21, and X19.

Principal component 4 is named wisdom by X14, X6, X16, X3, and X17.

Principal component 5 is named as syngroup by X11, X18, and X20.

Principal component 6 is named benevolence by X13 and X25.

### The Correlation Between Positive Quality and Responsibility for Innovation and Entrepreneurship

#### Spearman Correlation Analysis of Positive Psychological Quality and Responsibility of Innovation and Entrepreneurship

Table 10 reveals that there is a positive correlation between positive quality and responsibility for innovation and entrepreneurship as a whole, in which the correlation between positive quality and team responsibility and individual responsibility of college students reaches a significant level. Besides, in terms of positive quality, there is a positive correlation between justice dimension and social responsibility, but it does not reach a significant level; other quality and social responsibility reach a significant level, and the above correlation is significant. In Table 10, the highest correlation coefficient between intelligence and social responsibility was 0.662. In the positive quality group, the correlation coefficient between benevolence quality and other qualities is the highest, indicating that the more benevolent the quality of college students is, the more gregarious they are. In the group and other qualities, the correlation coefficient between benevolence quality and bravery quality is the highest, indicating that the more gregarious the college students are, the braver they are; compared with other qualities, bravery is higher than excellent quality, indicating that the braver the college students are, the more excellent their restraint is compared with other qualities, and the highest correlation coefficient with wisdom, indicating that the more restrained the college students are, the more intelligent they are. In the dimension of sense of responsibility, the correlation coefficient between team responsibility and social responsibility is as high as 0.7, indicating that the stronger the team responsibility is, the stronger the sense of social responsibility.

**TABLE 10:** Correlation analysis between positive quality and responsibility for innovation and entrepreneurship.

	Benevolence	Syngroup	Excellence	Bravery	Restraint	Wisdom	Team responsibility	Social responsibility	Individual responsibility
Benevolence	1								
Syngroup	0.271**	1							
Excellence	0.128**	0.198**	1						
Bravery	0.215**	0.248**	0.537**	1					
Restraint	0.174**	0.114**	0.339**	0.413**	1				
Wisdom	0.199**	0.222**	0.516**	0.513**	0.506**	1			
Team responsibility	0.250**	0.238**	0.556**	0.613**	0.509**	0.639**	1		
Social responsibility	0.181**	0.227**	0.476**	0.537**	0.472**	0.662**	0.700**	1	
Individual responsibility	0.156**	0.228**	0.628**	0.479**	0.328**	0.434**	0.606**	0.614**	1

**Correlation is significant at the 0.01 level (2-tailed).

#### Regression Analysis of Positive Psychological Quality and Responsibility of Innovation and Entrepreneurship

According to the results of linear regression in Table 11, the correlation coefficient (0.030 is significantly greater than *p* < 0.001) shows that under the condition that other variables remain unchanged, the positive psychological quality of college students increases by 1 unit, and the innovation and entrepreneurship responsibility increases by 0.030 units. The adjusted *R*^2^ shows that the regression model of positive psychological quality and team responsibility can explain 56.8% variance of college students’ innovation and entrepreneurship responsibility, the regression model of positive psychological quality and social responsibility can explain 51.0% variance of college students’ innovation and entrepreneurship responsibility, and the regression model of positive psychological quality and self-responsibility can explain 43.5% of college students’ innovation and entrepreneurship responsibility. Generally speaking, positive psychological quality has a higher explanation of team responsibility, followed by social responsibility, and the worst explanation of self-responsibility. In the linear fitting of positive psychology and team responsibility, benevolence, excellence, bravery, moderation, and wisdom all reached a significant level, among which the highest coefficient of knowledge and wisdom reached 0.307, indicating that for each increase of knowledge and wisdom, team responsibility increased by 0.307. In the positive psychology and social responsibility, the linear fitting of gregarious, excellent, brave, moderate, and wisdom reached a significant level; the highest intelligence coefficient was 0.463, indicating that for every 1-point increase in wisdom, it increased by 0.463; in the linear fitting of positive psychology and self-responsibility, gregarious, excellent, brave, moderate, and wisdom reached a significant level, of which the highest excellent coefficient was 0.526. It shows that for every 1-point increase in the excellent coefficient, the sense of self-responsibility increases by 0.307. In general, the regression coefficients of positive psychology, team responsibility, social responsibility, and personal responsibility are all greater than 0, which further shows that positive psychological quality has a positive impact on innovation and entrepreneurship responsibility (Burrell, 2020).

**TABLE 11:** Linear regression analysis of positive psychology to sense of responsibility.

	Team responsibility	Social responsibility	Individual responsibility	VIF (variance inflation factor)
Benevolence	0.054**	0.003	0.018	1.123
Syngroup	0.023	0.038*	0.062**	1.142
Excellence	0.177**	0.09**	0.526**	1.587
Bravery	0.269**	0.206**	0.161**	1.671
Restraint	0.139**	0.115**	0.061**	1.414
Wisdom	0.307**	0.463**	0.071**	1.76
Adjusted *R*^2^	0.568	0.510	0.435	

*Indicates p < 0.05, while ** indicates p < 0.001.

## Discussion

### Theoretical Meaning

The results of this study contribute to the theory of college students’ sense of responsibility. This study first found that in demographic variables, individual responsibility, social responsibility, and group responsibility were significantly different at the grade level; the personal responsibility of fourth*-*grade students was significantly higher than that of other grades; and the personal responsibility of third*-*grade students was significantly higher than that of first- and second-grade students (*p* < 0.05), which was similar to the results of Inada (2020). It showed that with the advancement of university life, individual college students were gradually maturely affected by the school education environment, which made their level of innovation and entrepreneurial responsibility significantly improved. Regardless of whether they were the only child or not, individual responsibility was not significantly different in gender (*p* > 0.05), while social responsibility and group responsibility were significantly different in gender level (*p* < 0.05). This may have resulted from the influence of traditional domestic education, in which men were still the main labor force, so their social responsibility also was heavy. This may be the additional responsibility given by the role. In addition, there was no significant difference in the sense of responsibility for entrepreneurial innovation between the only child level (*p* > 0.05), which was exactly the opposite of the results of Luo’s (2019) research. The reason of this may be that whether the child has responsibility comes from parents’ and families’ education and training concepts. So as long as the parent’s education is appropriate, regardless if they are an only child, they can develop a sense of social and group responsibility. Therefore, entrepreneurship is the highest form of employment, and responsible entrepreneurs are the goal of innovation and entrepreneurship education for college students. To enhance college students’ sense of responsibility for innovation and entrepreneurship is to let college students establish correct values in the activities of innovation and entrepreneurship and consciously achieve the unity of knowledge and practice. Innovation and entrepreneurship responsibility induction is an important part of ideological and political education for college students. This study will help to improve the content of ideological and political education for college students and enrich the theory of responsibility for college students.

Secondly, from the perspective of positive psychology, it was found that the linear fitting of benevolence, super-excellence, bravery, restraint, and wisdom and team responsibility all reached a significant level, and the coefficient of wisdom was the highest (0.307), which indicated that wisdom had the highest impact on the improvement and development of social responsibility of college students in positive psychological qualities, while benevolence, excellence, bravery, restraint, and wisdom had a significant predictive effect on group responsibility. Syngroup, excellence, bravery, modesty, and the linear fit of wisdom and social responsibility reached a significant level, with the highest coefficient of wisdom (0.463). This also showed that syngroup, excellence, bravery, modesty, and wisdom had a significant predictive effect on social responsibility. The linear fitting of syndication, excellence, bravery, gentleness, and wisdom to personal responsibility reached a significant level, with the highest coefficient of excellence (0.526), and other studies, indicating that syngroup, excellence, bravery, modesty, and wisdom had a significant predictive effect on group responsibilities, of which excellence had the highest impact on the improvement and development of college students’ social responsibility.

This paper explores the impact of positive psychological quality on college students’ sense of responsibility for innovation and entrepreneurship. Previous studies focused on the analysis and interpretation of relevant theories from the perspective of positive psychology, exploring how to improve the research of college students’ willingness to innovate and entrepreneurship and innovation and entrepreneurship education, focusing on the analysis and interpretation of relevant theories. This study through empirical research, through statistical analysis of positive psychological quality and college students’ sense of responsibility for innovation and entrepreneurship, broadens the research perspective of positive psychological quality theory.

Finally, the authors hope to make new theoretical contributions by comparing the significant correlation patterns of emotional feedback and cognitive feedback. According to the results of this survey, the cultivation system of college students’ sense of responsibility for innovation and entrepreneurship is proposed from the perspective of positive psychology. Positive psychology clearly points out that the existence of environment provides the possibility for human beings to show their own experience, and human beings will be influenced by the environment. Positive psychology refers to a wide range of environments, not only involving the material environment, but also including social, school, and family cultural environment. Based on this, in order to create a harmonious and orderly learning environment for students and effectively improve their sense of responsibility for innovation and entrepreneurship, the colleges and universities may choose to work from the following aspects.

#### Create a Positive Cultural Atmosphere

In practical work, it is necessary to put an end to the wrong practices that only stay in management, go deep into teachers and students, actively carry out publicity and education, carry out psychological education through multiple channels, and implement the multiple links of entrepreneurship and innovation. (1) Through observation and learning, students can be organized to watch publicity and education films or biographies of successful people or to experience the entrepreneurial enthusiasm of entrepreneurs in the innovation and entrepreneurship base, forming a resonance and then embarking on the road of entrepreneurship (Zhou, 2009). (2) Speech incentive: The colleges and universities may invite entrepreneurs who have successfully started their own businesses to give speeches and attract students through rich entrepreneurial experience. Under the guidance of an example, college students will become more ambitious and form a spiritual quality that sticks to the end and never advances (Zhang, 2017). (3) Communication and exchange: Through the organization of entrepreneurship exchange meeting and other forms, an exchange platform should be provided to the entrepreneurial students (Wang, 2015). Through this platform, students can exchange entrepreneurial experience and confusion. With the careful answers of entrepreneurial teams, the sense of panic caused by the insufficient understanding of entrepreneurial innovation will also disappear. (4) Creating entrepreneurial plans and organizing college students to implement the conception and creation of entrepreneurial plans: Through this action, students’ internal thoughts can be fully expressed, and the transformation from simple thoughts to practical actions can be realized, and the unity of thoughts and actions in a real sense can be realized.

#### Building a First-Class Team of Entrepreneurial and Innovative Teachers

At present, the primary task for colleges and universities is to establish a full-time teacher team to adapt to the changes. The teachers in the team should not only have the sense of responsibility for entrepreneurship education but also master the advanced teaching concepts and scientific methods. First of all, in order to build a team of high-quality college students’ innovation and entrepreneurship guidance teachers, it is necessary to strengthen the vocational training of the guidance teachers, optimize the psychological knowledge structure of the guidance teachers, and make them become high-quality guidance teachers with broad knowledge, solid professional foundation, rich practical experience, and the combination of “Tao” and “Shu” (Wang, 2003). Secondly, it is feasible to take the Chinese traditional culture as the philosophy guidance. In order to help students, understand and accept themselves, improve the positive personality of entrepreneurs and innovators, and stimulate the innovative thinking of college students, it is essential to actively adopt immersive auxiliary working methods; to adhere to the principles of curriculum design of preaching, teaching, and puzzle solving; and to create a good atmosphere full of love and sincerity for students. Finally, in the process of guiding the innovation and entrepreneurship practice of college students, the authors can adopt such methods as inspiring learning, simulating practice, exploring problems, and sharing experience, so as to make ourselves and students be full of positive emotions in the process of entrepreneurship practice; make practice teaching more targeted, happy, positive, and substantial in practice; and require the innovation and entrepreneurship tutors to pay attention to their own cultivation and maintain optimistic management quality.

#### Building a Scientific Curriculum System

The contents and methods of college students’ study in school depend on the construction of curriculum system, especially in stimulating students’ positive energy and cultivating positive emotions. The construction of curriculum system with positive psychological content plays an important role in this regard. First of all, the mental health knowledge is flexibly applied to the cultivation of students’ psychological quality. Secondly, in the curriculum system of mental health education and innovation and entrepreneurship, colleges and universities should actively cultivate and strengthen the internal positive characteristics of human beings, change, or even eliminate the negative characteristics (Li et al., 2019). Therefore, the content of positive psychological quality education can be added in the curriculum system of innovation and entrepreneurship of college students, and focus should be on the mental health education of entrepreneurs and innovators from the negative to the accumulation extreme. Finally, in terms of specific curriculum, the colleges and universities should appropriately increase the content of courses to meet the needs of students according to their actual needs (Chen, 2019). Since constructiveness is the essence of curriculum content, the teachers, students, the developers of curriculum, and content may work together to measure and develop curriculum and make constant evaluation and modification, so as to better play the role of college students’ positive psychological quality in the cultivation of innovative entrepreneurs’ sense of responsibility.

## Conclusion

This study explores the current situation of college students’ sense of responsibility for innovation and entrepreneurship, which provides some numerical references for colleges and universities, so that colleges and universities may think about college students’ innovation and entrepreneurship education from other perspectives. Through the analysis, it has been proved that the positive psychological quality can help enhance college students’ sense of responsibility of innovation and entrepreneurship. Therefore, college students may form a positive psychological quality through adequate psychological education, so that they can get a positive emotional experience in campus. Students should not be afraid of psychological counseling (Qian et al., 2018). In short, academic innovation and entrepreneurship had significant differences in gender and grade levels. Positive psychological qualities such as syngroup, excellence, restraint, benevolence, super-excellence, bravery, restraint, and wisdom were the factors that influence college students ’sense of responsibility for innovation and entrepreneurship. Universities should devote themselves to creating a cultural atmosphere of innovation and entrepreneurship in order to form a perfect curriculum system, inspire students ’sense of responsibility for innovation and entrepreneurship, and participate in innovation and entrepreneurship.

### Limitations and Future Research

The results of this study should be interpreted with caution. First of all, as the authors have conducted this research in China, it is unclear how many research results can be extended to the Western countries. In China, social responsibility is a relatively important content, which is related to China’s system. China is a society guided by the sense of collective honor. For the requirements of college students’ innovation and entrepreneurship, it is particularly important to pay attention to the social value of the industry, so college students are also required to shift their attention to the sense of social responsibility. Therefore, it is suggested that in the future research, more attention should be paid to exploring the influence degree and model theory of positive psychological quality on the responsibility of innovation and entrepreneurship according to the actual requirements of various countries.

Secondly, in the discussion of this study, the authors only analyzed the correlation significance and linear regression model about the influence of positive psychological quality on the innovation and entrepreneurship responsibility of college students and did not explore the structural equation model in depth. At the same time, this study did not examine the intermediary factors of positive psychological quality on the innovation and entrepreneurship responsibility of college students. As a result, the authors expect that the role of mediators may be clarified in the future research.

## Data Availability Statement

The raw data supporting the conclusions of this article will be made available by the authors, without undue reservation, to any qualified researcher.

## Ethics Statement

The studies involving human participants were reviewed and approved by the Nanjing Xiaozhuang University Ethics Committee. The patients/participants provided their written informed consent to participate in this study. Written informed consent was obtained from the individual(s) for the publication of any potentially identifiable images or data included in this article.

## Author Contributions

All authors listed have made a substantial, direct and intellectual contribution to the work, and approved it for publication.

## Conflict of Interest

The authors declare that the research was conducted in the absence of any commercial or financial relationships that could be construed as a potential conflict of interest.
